# Endoplasmic reticulum homeostasis—From molecules to organisms: Report on the 14th International Calreticulin Workshop, Saint Malo, France

**DOI:** 10.1111/jcmm.17840

**Published:** 2023-07-06

**Authors:** Ketsia Bakambamba, Federico Di Modugno, Marianne Guilbard, Simon Le Goupil, Stephanie Lhomond, Diana Pelizzari‐Raymundo, Tony Avril, Eric Chevet, Frédéric Delom, Elodie Lafont

**Affiliations:** ^1^ Inserm U1242 University of Rennes Rennes France; ^2^ Centre de Lutte Contre le Cancer Eugène Marquis Rennes France; ^3^ Inserm U1312, ARTiSt Lab University of Bordeaux Bordeaux France; ^4^ Thabor Therapeutics Paris France

**Keywords:** AGR2, cancer, chemotherapy, Calreticulin, degenerative diseases, endoplasmic reticulum, ER stress, immune regulation, unfolded protein response

## Abstract

The Calreticulin Workshop, initiated in 1994 by Marek Michalak in Banff (Alberta, Canada), was first organized to be an informal scientific meeting attended by researchers working on diverse biological questions related to functions associated with the endoplasmic reticulum (ER)‐resident lectin‐like chaperone and applied to a wide range of biological systems and models. Since then, this workshop has broadened the range of topics to cover all ER‐related functions, has become international and has been held in Canada, Chile, Denmark, Italy, Switzerland, UK, USA, Greece and this year in France. Each conference, which is organized every other year (pending world‐wide pandemic), generally attracts between 50 and 100 participants, including both early career researchers and international scientific leaders to favour discussions and exchanges. Over the years, the International Calreticulin Workshop has become an important gathering of the calreticulin and ER communities as a whole. The 14th International Calreticulin Workshop occurred from May 9–12 in St‐Malo, Brittany, France, and has been highlighted by its rich scientific content and open‐minded discussions held in a benevolent atmosphere. The 15th International Calreticulin Workshop will be organized in 2025 in Brussels, Belgium.

## 1 INTRODUCTION

**FIGURE 1:**
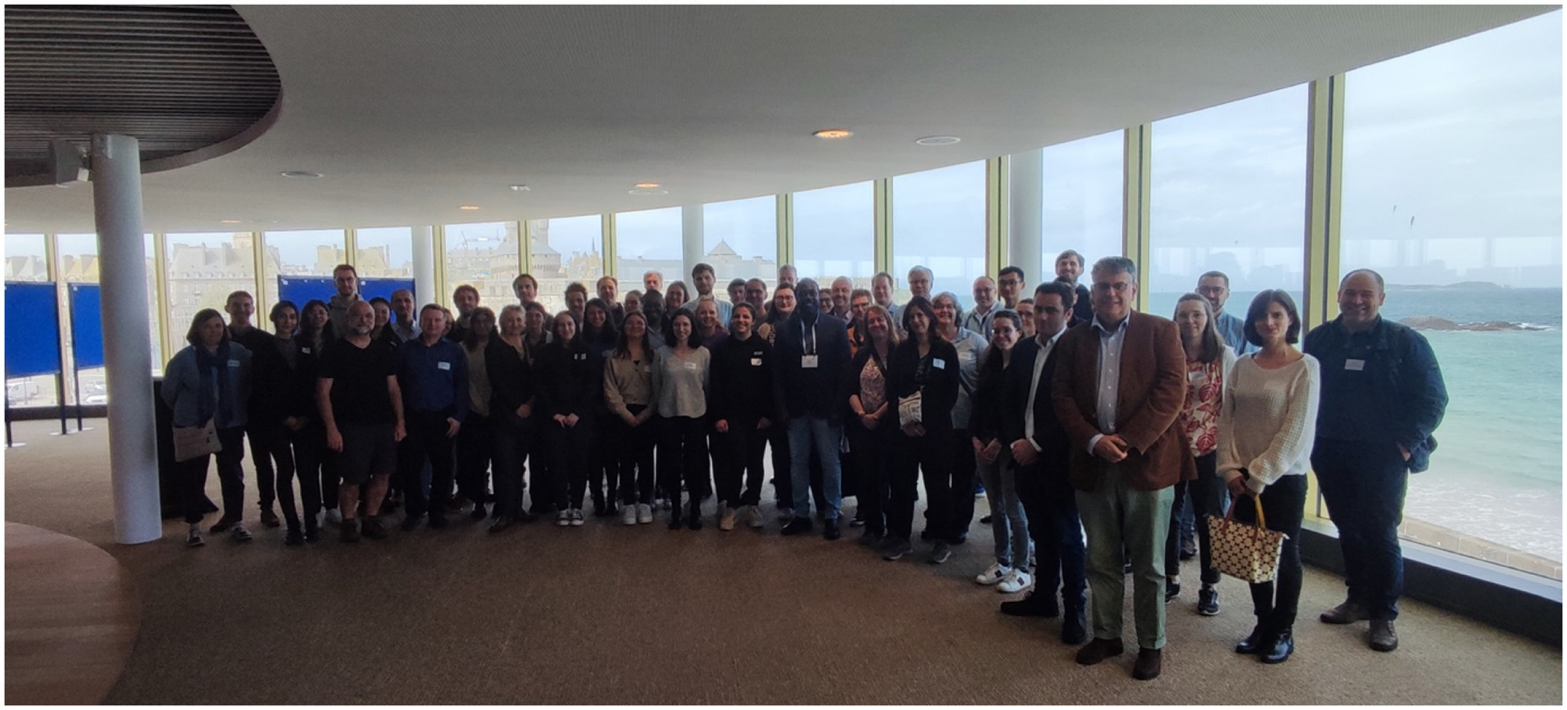
Picture of the 14th International Calreticulin Workshop participating members in the Rotonde at the Palais du Grand Large in Saint Malo with a fantastic background.

An international workshop dedicated to the study of calreticulin and other endoplasmic reticulum (ER) chaperones has been taking place every 2 years since 1994. The Calreticulin workshop derives its name from the calreticulin protein (CALR), an ER‐resident lectin‐like chaperone initially identified in 1974 as a high‐affinity Ca^2+^ binding protein in the muscle sarcoplasmic reticulum.^1^ With a profound interest in this protein and the belief in its fundamental role within the ER, Pr. Marek Michalak organized the inaugural International Calreticulin Workshop in Banff, Canada, thus marking the beginning of a biannual tradition. Subsequent workshops were hosted in various countries, including Italy, Switzerland, the USA (twice), the UK, Chile, Canada (four times), Denmark and Greece. Since the first meeting, the workshop emphasized the discussion on the structure and function of the ER, as well as the protein/lipid quality control mechanisms.^2^, ^3^, ^4^ After 29 years, the 14th meeting took place for the first time in France in Saint Malo, May 9–12, 2023 (Figure 1). The workshop hosted researchers from 12 different countries mostly located in North and South America, Middle East and Europe. They presented their latest findings and engaged in extensive discussions with fellow colleagues. The presentations covered a broad range of ER‐related topics, including the molecular mechanisms of secretory autophagy, the role of Anterior GRadient proteins, structural characteristics of calreticulin and its functional aspects in immunity and diseases, the impact of ER stress and the unfolded protein response (UPR) in cancer and neurodegenerative diseases, and novel mechanisms controlling the functions of the early secretory pathway. A significant focus of the workshop regarded the relationship between ER biology and various diseases. This included many types of cancers (e.g. blood, brain, breast, colorectal, pancreas and lung cancers), degenerative diseases (such as age‐related macular degeneration (AMD), Alzheimer's and Parkinson's diseases, multiple sclerosis, amyotrophic lateral sclerosis, Wolfram syndrome) and COVID‐19 (see sections below). Collectively, this edition of the International Calreticulin Workshop presented a vision of ER functions at multiple scales from the molecule (e.g. AGR2, CALR and Spike) to the cell (e.g. protein secretion, calcium homeostasis and translation) and at last evaluated their impact in diseases such as cancer or degenerative disorders. Of note, therapeutically actionable mechanisms related to ER functions were also described in a number of presentations thereby highlighting the enormous potential of exploring ER biology beyond fundamental science.

The Keynote lecture was given by Dr Thierry Galli (Inserm, Paris, France) on the role of unconventional protein secretion (UPS) in brain diseases. The UPS allows the secretion of cargos lacking a signal peptide or proteins that manage to escape the Golgi apparatus despite entering the ER. Cargos subjected to UPS can be further classified into different types based on their mode of transport. Types I and II are independent of vesicles or SNARE proteins, while Types III and IV do rely on those. VAMP7, a vesicular SNARE, plays a significant role in exosome, lysosome, and autophagy‐mediated secretion and is an essential part of axon growth fine‐tuning. This mechanism is partly dependent on the secretion of VGF (not an acronym, rather ‘Victory’ Growth Factor) or pro‐VGF through the UPS. Pro‐VGF appears to be associated with extracellular vesicles (EVs) and endo‐lysosomes. Moreover, the overexpression of LRRK2 disrupts the peripheral localization of VGF in primary neurons. Taken together, these findings suggest that LRRK2 regulates the secretion of VGF/pro‐VGF by interacting with VAMP7, thus influencing its trafficking and release.

## 2 SESSION I—AGR/PDI

The first session aimed to clarify the functions of Protein Disulfide‐Isomerase (PDIs) with a specific focus on the Anterior GRadient (AGR) protein family. There is a growing interest in understanding the novel functions acquired by the three members AGR1‐3 in many diseases. It is well established that AGR proteins are ER‐resident, showing both N‐terminal signal peptide and C‐terminal pseudo‐KDEL, with foldase/holdase and ER protein homeostasis maintenance functions. AGR proteins display variable tissue expression patterns, and their expression is regulated by different signalling mechanisms. While primarily localized to the ER, AGR proteins have also been detected in other cellular compartments such as the cytosol and extracellular environment where they exert non‐canonical gain of functions. Among the AGR proteins, AGR2 has been the most studied member. It has been found to be overexpressed in most epithelial cancers, which generally correlated with poor patient outcome.^5^ ER‐associated functions of AGR proteins were discussed by Dr Arvin Pierre (Glasgow University, Glasgow, UK) who highlighted the importance of AGR1 (ERp18) in the ER as a regulator of the activation of the UPR sensor ATF6α (hereafter referred to as ATF6).^6^ ATF6 luminal C‐terminal region contains two cysteine residues (467 and 618) able to form either intra‐ or inter disulfide chains. In unstressed cells, ATF6 predominantly exists as a monomer with some disulfide‐linked oligomers detected. Upon stress, the C467‐C467 interchain dimer is increased while the C467‐C618 intrachain form is decreased, giving rise to the suggestion that only this dimeric form exits the ER towards the Golgi. In accord, the cleaved ATF6 luminal domain is a dimer. Of note, ERp18 controls ATF6 redox status, and thus its dimerization. Indeed, ERp18 selectively associates with ATF6 upon stress, reduces interchain disulfide and thus ERp18 overexpression impairs ATF6 trafficking to the Golgi. The interaction between ERp18 and ATF6 may prevent the premature exit from the ER in the absence of stress. Dr Roman Hrstka (Masaryk University, Brno, Czech Republic) focused on investigating the AGR2 interactome in breast cancer models. Using reversible crosslinking followed by a pull‐down of AGR2 complexes and high‐resolution LC–MS/MS, the team identified a complex comprising AGR2, PDIA3 and PDIA6, which was further confirmed through co‐immunoprecipitation.^7^ Induction of ER stress in breast cancer cells enhanced the AGR2‐PDIA3 complex formation and increased extracellular levels of AGR2, thus suggesting that the AGR2‐PDIA3 interaction might contribute to AGR2 secretion. Then Dr Delphine Fessart (Inserm, Bordeaux, France) presented evidences revealing the involvement of extracellular AGR2 (eAGR2) in tumorigenic processes, prompting her group to explore the role of eAGR2 in cell plasticity. She demonstrated that eAGR2 represents a pro‐oncogenic regulator of epithelial morphogenesis and tumorigenesis^8^; and that AGR3 (which presents ~70% homology with AGR2) also shows the ability to promote cell migration in cell culture models.^9^ Dr Fessart also discussed the role of AGR2 in chemoresistance demonstrating that Doxorubicin‐resistant tumour cell lines (MCF7‐R) displayed increased expression and secretion of AGR2. To overcome this resistance, the addition of an AGR2 blocking antibody to the culture media sensitized resistant tumour cells to chemotherapeutic treatment, suggesting that targeting eAGR2 within the tumour microenvironment might represent a promising strategy to overcome chemotherapy resistance. Dr Aeid Igbaria (Ben Gurion University, Beer Sheva, Israel) focused on the reflux of ER‐resident proteins to the cytosol, a mechanism known as ER to CYtosol Signaling (ERCYS^10^). In stressed cells, as well as in cancer cells, PDIA1, PDIA3 and AGR2 were found to be present in the cytosol. Provided that cytosolic AGR2 (cAGR2) inhibits p53 and that cytosolic PDIA1 (cPDIA1) can interact with the cleaved form of Caspase 3 (ongoing studies on the putative biological effects of the interaction on apoptosis), this might indicate that cytosolic PDIs could contribute to stress resistance mechanisms commonly observed in chemoresistant cells. In an effort to characterize the molecular mechanisms underlying ERCYS, Dr Igbaria identified HSC70 and DNAJB12 as key players in this process. Dr Olivier Pluquet (University of Lille, Lille, France) discussed the involvement of AGR2 in gastroesophageal junction (GEJ) adenocarcinoma chemoresistance. The analysis of healthy tissues, as well as pre‐and post‐treatment tumour samples, unveiled the upregulation of several ER‐related genes in chemotherapy‐treated samples, among which AGR2 was identified. Notably, low expression of AGR2 was associated with a more favourable response to treatment. Thus, these findings raised the question of whether AGR2 could sensitize tumour cells to chemotherapy. Importantly, AGR2 was silenced in OE19 cells (adenocarcinoma of gastric cardia/oesophageal gastric junction) which resulted in increased sensitivity to chemotherapies. These findings indicate that reducing the expression of AGR2 could potentially improve the responsiveness of tumour cells to chemotherapy. Lastly, Andrea Martisova (Masaryk University, Brno, Czech Republic) presented data on the implication of AGR2 in epithelial‐to‐mesenchymal transition (EMT). A transcriptomic approach in lung cancer cells (A549) knocked‐out for AGR2 or not and treated or not with TGF‐β treatment unveiled significant changes in transcripts related to focal adhesions and arachidonic acid metabolism, two pathways involved in EMT. Notably, the knockdown of AGR2 led to a significant decrease in PGE2 biosynthesis whereas PGE2 addition resulted in an upregulation of AGR2 expression.^11^ These results establish a connection between AGR2 expression, arachidonic acid metabolism and EMT in lung cancer cells.

## 3 SESSION II—CALRETICULIN (SFC SESSION)

Many presentations in this session, sponsored by the Société Française de Cancer, focused on the structural and functional aspects of calreticulin (CALR) mutants in myeloproliferative neoplasms (MPN).^12^ Pr Stefan Constantinescu and Mr Nicolas Papadopoulos, PhD Student (Ludwig Institute for Cancer Research and University of Louvain, Belgium) discussed the properties of a class of frameshift mutations in CALR, with a prevalent exemplar, namely CALR del52 (deletion of 52 bp), occurring in *JAK2* V617F‐negative and *MPL/TpoR* mutant‐negative MPNs. Hydrogen‐deuterium exchange mass spectrometry (HDx‐MS) analysis showed that CALR del52 exhibited a global modification of its conformation, resulting in increased accessibility for its chaperone role. Remarkably, changes in the C‐terminal domain of CALR induced by the del52 mutation led to an alteration in the structure of the N‐terminal domain. This alteration is of importance as it unmasks the N‐glycan binding domain, enhancing the binding capacity of immature N‐glycans, thus conferring the ability of CALR del52 to further bind to the luminal/extracellular domain of the Thrombopoietin‐Receptor (Tpo‐R). Data also showed that mutant CALRs are secreted from clonal cells that do not express TpoR and the plasma form behaves like a cytokine, oligomerizing with endogenous mutant CALR at the surface of TpoR expressing cells; in this way the circulating form activates the TpoR on cells belonging to the MPN clone.^13^ The specificity of binding to the TpoR extracellular domain comes from interactions between the positively charged repetitive sequences in the new tail of mutant CALR and several negatively charged patches on TpoR D1 domain.^14^ This CALR mutant binding to Tpo‐R activates the receptor, thereby acting as a cytokine to sustain aberrant proliferation of haematopoietic cells. These findings shed light on the mechanistic role of CALR del52 in promoting the aberrant proliferation of haematopoietic cells through its interaction with Tpo‐R. Dr Malini Raghavan (University of Michigan, MI USA) further described the role of CALR mutations in cell transformation focusing on the relevance of the novel C‐terminal domain of CALR mutants in TPOR binding and the different modes of mutant CALR multimerization relevant to Tpo‐R activation.^15^ In this context, enhanced degradation of CALR mutants could lead to reduced cell proliferation in some MPN subtypes. Remarkably, lysosomal pathways synergistically increased mutant CALR and Tpo‐R degradation, and their induction decreased cell proliferation induced by mutant CALR in cell lines and proliferation of CD34 positive cells from MPN patients. Next, Dr Ann Mullaly (Harvard University, Boston, USA) continued on CALR mutation, by examining CALR del52 and the epigenetic regulator ASXL1 (Additional Sex Combs Like Transcriptional Regulator 1, a protein that functions as a ligand‐dependent co‐activator for retinoic acid receptor in cooperation with nuclear receptor co‐activator 1). Whereas CALR del52 is a common mutation in MPN patients, ASXL1 mutations arising in myelofibrosis reduce the survival of CALR del52 patients. This was confirmed using CALR del52/ASXL1^mut^ mice which exhibited a more severe MPN phenotype and aberrant megakaryocyte profile compared to CALR del52 MPN. At the molecular level, ASXL1 mutation led to a gain of function of the ASXL1‐BAP1 deubiquitination complex and an increase in Histone H3 Ser10 phosphorylation, a marker of proliferation. The subsequent two presentations focused on calreticulin's role in immunity. Macrophage phagocytosis requires emission of signals by apoptotic or cancer cells to initiate programmed cell removal. CALR was long thought to be a pro‐phagocytic signal expressed on the surface of cancer cells. Allison Banuelos (Stanford University, CA USA) described the secretion of CALR by macrophages which binds to asialoglycans on target cells, providing the ‘eat me’ signal for phagocytosis. Investigating the aberrant secretion of CALR containing a KDEL ER‐retention motif, she demonstrated that TLR activation played a role in CALR translocation and subsequent secretion. This was further discussed by Allison Zhang (Stanford University, CA USA) who specified that CALR secretion by macrophages was associated with phagocytic activity and that both were higher in M1 macrophages than in M2. The last presentation of the session focused on age‐related macular degeneration (AMD), which causes a blurry central vision. In the past years, the prevalence of AMD has been increasing, and the current standard treatment, VEGF intravitreal injection, is invasive and may cause side‐effects. Dr Rimantas Slibinskas (Vilnius University, Vilnius, Lithuania) discussed topical applications of CALR as a potential therapeutic strategy against AMD using laser‐induced choroidal neovascularization (CNV) models. This relies on the anti‐angiogenic properties of CALR fragment vasostatin (corresponding to the 180 N‐terminal amino‐acids of CALR).^16^ Mice developing CNV were treated by either intraocular injection or topical application of full‐length CALR, consisting in application of drops able to penetrate into the eye. In vivo angiography demonstrated that both application methods effectively inhibited angiogenesis and slowed disease progression, although topical application showed a delay in resolving vascular lesions. Remarkably, CALR topical applications at lower doses were more effective than higher doses, suggesting a dose‐dependent effect and the possibility of off‐target effects at high concentrations. Furthermore, the physico‐chemical analysis of the CALR drop solution indicated high stability at room temperature and below (whereas instability was observed at 37°C), which could be of interest to control the bioavailability of the protein. However, the precise mechanism of action by which CALR inhibits angiogenesis in vivo still remains unclear.^17^

## 4 SESSION III—CANCER (FONDATION ARC SESSION)

This third session, sponsored by the Fondation ARC pour la Recherche sur le Cancer, started with a presentation on protein translocation in the ER as one of the early steps of canonical protein secretion and as such, is an important component of ER proteostasis in cancer. Dr Caroline Demangel (Pasteur Institute, Paris, France) studies an infectious disease caused by *Mycobacterium ulcerans*, leading to ulcer development. This bacterium secretes the mycolactone polyketide, a non‐selective inhibitor of the Sec61 translocon that prevents nascent proteins from entering the ER.^18^ This inhibition potently affects IFN‐inducible proteins, such as cytokines/chemokines and their receptors (e.g. IL‐6R downregulation affecting IL‐6 signalling pathway), TCR response, and antigen presentation, overall leading to an immunosuppressive effect of mycolactone. Taken together, these observations led to the consideration of mycolatone for therapeutic use. In vivo experiments have confirmed that both mycolactone and a synthetic derivative protect against chronic skin inflammation and inflammatory pain. Additionally, mycolactone synergizes with proteasome inhibitors and immunomodulators (such as Lenalidomide) in multiple myeloma cellular and mouse models, enhancing cell death likely through terminal UPR signalling. Next, Dr Jean‐Ehrland Ricci (Inserm, Nice, France) presented new insights into metabolic effects on anti‐tumour immunity. Using mouse models with colon cancer xenografts, it was observed that a 25% reduction in protein consumption (but not carbohydrates) led to reduced tumour growth upon treatment with chemotherapy. However, this effect was not observed in mice deficient in CD8+ T cells, indicating that a low protein diet promotes anti‐tumor immunity through at least the activation of T cells. Further investigations revealed that a low protein diet activates the IRE1 branch of the UPR in tumour cells and that IRE1 inhibition reduced the benefits from the diet. Conversely, IRE1 overexpression in colon cancer cells resulted in reduced tumour progression and increased tumour‐infiltrating lymphocytes, thus providing an anti‐tumour role of IRE1, which triggers the immune response based on metabolic cues. An in situ interactome analysis of IRE1 identified a component of a mannose‐Transferase complex responsible for providing Mannose substrate for N‐glycosylation. This protein is overexpressed in colon adenocarcinoma. Cells KO for this gene exhibited increased oligomerization of IRE1 and hypoglycosylation of PD‐L1. As a result, tumour cells lose their ability to trigger this immune checkpoint. Analysis of immune infiltration revealed that KO tumours displayed less pro‐tumoral M2 macrophages (which is consistent with reduced display of mannose) and more CD3+/CD8+ cells. These findings link metabolic regulation to the N‐glycan biosynthesis and the subsequent ER stress regulation of tumour immune control. Next, Dr Jody Groenendyk (University of Alberta, Edmonton, Canada) pursued the discussion on cancer metabolism and presented data on fructose, one of the main sources of energy of cancer cells, transported by the SLC2A5 (GLUT5) transporter, often upregulated in cancer.^19^, ^20^ Using boyden chamber and scratch assays in pancreatic cancer cell lines knocked out for GLUT5, impaired cell migration was observed. However, this was rescued when full‐length GLUT5 was reintroduced, but not when a transport‐defective mutant was used, indicating that the impaired migration is likely due to fructose transport. In vivo experiments corroborated these results, with lower metastasis potential of GLUT5 knockout fibrosarcoma cells compared to wild‐type, accompanied by a decrease in primary tumour growth. Microscopy analysis revealed that GLUT5 knockout cells had a less pro‐migratory morphology with few protrusions and exhibited impaired mitochondria morphology and transport. Loss of GLUT5 thus prevents transport of mitochondria at leading edges of cells, where they are supposed to sustain migration. The last three presentations focused on glioblastoma (GB), a tumour associated with increased matrix stiffness within and around the tumour, which triggers signalling pathways through cytoskeleton rearrangement.^21^ Pr Frank Kruyt (University of Groningen, Groningen, Netherlands) presented data on the mechano‐transducer role of the UPR sensor, PERK. PERK KO in GB cell lines showed impaired cell adaptation to matrix stiffness due to a defect in F‐actin polymerization. These data linked PERK to its association with the actin‐binding protein filamin A (FLNA).^22^ By scaffolding FLNA, PERK induces remodelling of the actin cytoskeleton and promotes the formation of focal adhesions, which contributes to the adaptation of matrix stiffness. Continuation of these findings could lead to considering PERK as a relevant therapeutic target in GB treatment. This notion was supported by the presentation of Dong Liang (University of Groningen, Groningen, Netherlands), working on PERK modulation as a therapeutic tool for GB. Using the PERK activator MK‐28, GB viability was reduced, and cell cycle arrest in the G1 phase was observed. Another therapeutic approach in GB was presented by Dr Diana Pelizzari‐Raymundo (Inserm, Rennes, France) on her work regarding the development of a novel blood brain barrier (BBB) permeable IRE1 inhibitor. GB growth is supported by IRE1 activation, and the design of a BBB permeable IRE1 kinase inhibitor named Z4P inhibited IRE1 RNase activity. In vivo experiments confirmed the potential of this approach, since combination of these IRE1 inhibitors with temozolomide synergistically reduced GB tumour relapse. Currently, a structure–activity relationship study is ongoing to optimize physico‐chemical properties of the compounds, as well as to identify more potent analogues for inhibiting IRE1.

## 5 SESSION IV—DEGENERATIVE DISEASES

In this session, the intricate relationship between degenerative diseases and ER functions was examined. Dr Benjamin Delprat (Inserm, Montpellier, France) presented his work on Wolfram syndrome (WS), an autosomal recessive genetic disorder characterized by diabetes, optic nerve atrophy, deafness and neurodegeneration. The progression of the disease ultimately leads to premature death of the patients around 35 years of age. While WS has long been considered a mitochondrial disease, recent hypotheses suggest the involvement of the ER in the syndrome. Variants in the WFS1 gene, coding for the Wolframin ER Transmembrane Glycoprotein, lead to the synthesis of proteins localized to the ER, leading to WS type 1, which represents 99% of WS cases. WFS1 plays a crucial role in regulating the transfer of mitochondrial calcium through the mitochondria‐associated ER membranes (MAMs). The association of WFS1, NCS1 and the inositol‐1,4,5‐trisphosphate receptor (IP3R) prevents NCS1 degradation and sustains ER‐mitochondria calcium transfer. In WS, this calcium transfer is disrupted, and connections between the ER and mitochondria are reduced, a phenotype also observed upon NCS1 knockdown. The team then developed a zebrafish model of WS by disrupting both Wfs1a and Wfs1b genes and investigated the potential therapeutic effect of Ncs1 overexpression. Overexpression of Ncs1 using mRNA microinjection restored mitochondrial alterations and locomotor hyperactivity in the mutant larvae. Based on these results, the researchers propose Ncs1 as a potential therapeutic target for WS. The zebrafish model offers a useful platform for drug screening in WS and complements other animal models like rodents, in furthering our understanding of this disease. Dr Myriam Pujol (University of Alberta, Edmonton, Canada) gave a short talk on the interaction between calnexin (CANX) and fatty acid binding protein 5 (FABP5), which facilitates T‐cell movement through the BBB. These findings suggest that the regulatory protein CD200 plays a significant role in reducing T‐cell permeability in the absence of CANX or FABP5. These data highlight the potential of targeting this protein complex as a treatment approach for neuro‐inflammatory diseases. To conclude the session on degenerative diseases, Pr Claudio Hetz (University of Chile, Santiago, Chile) delivered a presentation on the pivotal role of PDIs in maintaining proteostasis in the nervous system. Specifically, Pr Hetz focused on the significance of PDIA3 in amyotrophic lateral sclerosis (ALS), a progressive paralytic disorder characterized by the selective degeneration of motor neurons in the brainstem and cerebral cortex. Overexpression of PDIA3 in neurons promotes axon elongation, while inactivating mutations of PDIA3 in zebrafish caused detrimental effects in the animals. The identification of a mutation in the catalytic site of PDIA3 (C57Y), associated with severe intellectual disability and neurodevelopmental problems in humans, led to its insertion in zebrafish and mouse models to study the associated pathogenic mechanisms. Zebrafish embryos carrying the mutation exhibited severely impaired development and abnormal morphologies. In mice, synaptic activity in the hippocampus was significantly impaired, resulting in altered neurogenesis and behavioural changes. Additionally, the C57Y PDIA3 mutants formed aggregates that abnormally interacted with the chaperones CANX and CALR. Moreover, the PDIA3‐C57Y mutant also affected the biogenesis and signalling of integrins by disrupting the actin cytoskeleton and impairing neuritogenesis. Biochemical investigations finally revealed that the mutant exhibited lower enzymatic activity and formed aggregates due to increased disulfide bonds.

## 6 SESSION V—ER FUNCTION (SBCF SESSION)

The last session of the workshop, sponsored by the Société de Biologie Cellulaire de France, focused on investigating other ER functions. The session was opened by Dr Laurence Abrami (EPFL, Lausanne, Switzerland) who introduced her work on SARS‐CoV‐2 and its impact on increased Spike protein S‐acylation (or S‐Palmitoylation), which enhances infectivity providing new information about infection mechanisms of the virus. S‐acylation is a post‐translational modification consisting in addition of an acyl lipid (composed of 12–20 carbons) to a cysteine residue through the action of enzyme ZDHHC S‐acetyltransferases. Spike protein S‐acylation stabilizes its structure. The efficiency of SARS‐CoV‐2 infection dropped upon S‐acylation mutants of Spike protein, suggesting that the S‐Acylation itself is necessary for stabilizing the Spike protein trimers. A siRNA screening of acyltransferase responsible for Spike protein S‐acylation unveiled four different enzymes, notably ZDHHC20 and ZDHHC9. Of note, SARS‐CoV‐2 infection resulted in the expression of a variant ZDHHC20 (upstream translation start site), leading to the production of a more stable enzyme that appears in stressed or infected cells only in the ER. In conclusion, SARS‐CoV‐2 could promote its own infective potential by triggering the expression of a more stable form of the enzyme responsible for Spike protein activation. Next, Andrew Bazley (ISG, NYU Langone Health, New York, NY USA) presented valuable insights into the regulation of protein diffusion in ER. The movement of particles within an environment is influenced by molecular crowding. In the specific context of ER lumen, the study of diffusion under different stimuli was made possible through the use of single particle tracking techniques, using ER‐genetically encoded multimeric nanoparticles. The diffusion of these particles upon ER stress varied depending on the stressor used. Higher diffusion rates were observed upon thapsigargin treatment (an ER calcium homeostasis disruptor) whereas lower diffusion rates were observed upon tunicamycin treatment (an N‐glycosylation inhibitor), suggesting that different causes of ER stress impact ER rheology. Ilaria Pontisso (Inserm, Saclay, France) presented the tight relationship between incremental ER calcium depletion and UPR activation. They combined experimental and mathematical modelling approaches to investigate how moderate calcium depletion can impact the activation of the different UPR sensor arms, to better understand the mechanisms underlying the progression of several diseases associated with ER stress. Treatment with tBubHQ (a reversible sarco/endoplasmic reticulum calcium‐ATPase (SERCA) inhibitor) resulted in BiP diffusion reduction, suggesting an accumulation of misfolded proteins in the ER, a condition known to activate UPR. This was consistent with a dose‐dependent phosphorylation of IRE1 and PERK, along with the accumulation of ATF6n (cleaved form of ATF6) in the nucleus. The computational model, supported by in vitro experiments, predicted the possibility of UPR reversion, which was indeed observed. Dr Alison Forrester (University of Namur, Namur, Belgium) presented her work on ER export and its relationships with autophagy. Indeed, autophagy is required for efficient ER‐Golgi trafficking, and it has been shown to promote the degradation of intracellular procollagens, to prevent their accumulation in the ER. ER‐phagy of pro‐collagen type I and II requires the interaction of CANX with FAM134B (also known as Reticulophagy Regulator 1 (RETREG1) protein), leading to their association with the phagophore. Dr Forrester then described a new target in retrograde transport. Shiga toxin can promote haemolytic‐uremic syndrome through its trafficking in the ER using a mechanism of retrograde transport from the plasma membrane. Through screening, a small molecule named Retro‐2 was identified, which exhibits high specificity in targeting the ER exit site protein Sec16A. By targeting this protein, Retro‐2 slows down Shiga toxin retro‐transport, a mechanism that could easily be transferred to other toxin‐derived pathologies. The last presentation of the workshop was delivered by Dr Celine Philippe (Barts Cancer Institute, London, UK) introducing her work on UPR and how it can affect the Alternative Splicing Landscape. Both the transcriptome and translatome are shaped upon ER stress and UPR activation, even within the initial minutes. However, our understanding of these processes is limited due to their dynamics. To investigate the de novo translation of mRNA upon ER stress, O‐Propargyl puromycin labelling of nascent peptides was used, followed by LC–MS/MS analysis. This approach revealed an enrichment of splicing factors, indicating their preferential translation in response to ER stress. Among the three arms of the UPR response to stress, PERK, was identified as responsible for the ER alternative splicing signature. The mechanisms by which these splicing events occur depend on the increased phosphorylation of specific mRNA binding proteins.

## 7 POSTER SESSIONS

During this event, two engaging poster sessions were conducted, featuring a diverse range of topics consistent with the sessions presented above. Over 15 poster presenters provided a concise overview of their data and findings (Appendix A). The posters covered a wide array of subjects, ensuring a stimulating and varied scientific discourse throughout the event and evaluated by an independent jury composed of invited speakers. Following the conclusion of the poster sessions, a recognition ceremony took place, where prizes were awarded by the generous sponsors of the event. The SBCF (Société de Biologie Cellulaire de France) presented awards to Hussein Issaoui and Daniela Ricci for their contributions. In addition, the Company of Biologists Ltd awarded travel prizes to Jody Groenendyk, Marianne Guilbard, Nardin Georgeos, Jérôme Archambeau and Arvin Pierre in recognition of their presentations. Furthermore, Diana Pelizzari‐Raymundo and Manon Nivet were awarded with the SFC (Société Française du Cancer) and the FEBS journal prizes, respectively. The awards ceremony not only celebrated the diverse achievements but also highlighted the collaborative and supportive nature of the scientific community.

## 8 CONCLUSION

The 14th International Calreticulin workshop offered a fantastic platform for scientists from around the world to share their research and foster collaborations in understanding the complex biology of the ER and its implications in various diseases. Moreover, this workshop provided an ideal opportunity for scientists from diverse disciplines and different career stages to come together with a shared objective of advancing the understanding of the functions of CALR and other ER proteins as potential therapeutic targets. The 15th International Calreticulin Workshop will be organized in Brussels, Belgium, in 2025.

## AUTHOR CONTRIBUTIONS

**Ketsia Bakambamba:** Writing – original draft (supporting). **Federico Di Modugno:** Writing – original draft (supporting). **Marianne Guilbard:** Writing – original draft (supporting). **Simon Le Goupil:** Writing – original draft (supporting). **Stéphanie Lhomond:** Writing – original draft (supporting). **Diana Pelizzari‐Raymundo:** Writing – original draft (supporting). **Tony Avril:** Writing – original draft (lead); writing – review and editing (supporting). **Eric Chevet:** Writing – original draft (lead); writing – review and editing (lead). **Frederic Delom:** Writing – original draft (lead); writing – review and editing (supporting). **Elodie Lafont:** Writing – original draft (lead); writing – review and editing (supporting).

## CONFLICT OF INTEREST STATEMENT

EC is founder of Thabor Therapeutics.

## Data Availability

There is no data included in the article.

## APPENDIX A

**Table:**

Poster Session#1	
F. Di Modugno	AGR2 pro‐oncogenic gain‐of‐function associated with its non‐canonical localization: a role in cholangiocarcinoma
M. Guilbard	AGR2: A link between ER proteostasis and cancer proliferation
A. Pierre	Investigating the role of ERp18 during Activation of UPR sensor ATF6α
M. Baez	Endoplasmic reticulum‐to‐Golgi trafficking of Calreticulin is required for its translocation and secretion by macrophages
A. Banuelos	Investigation of Calreticulin processing and secretion by macrophages for programmed cell removal
N. Georgeos	Identification of Calreticulin‐binding partners on the cell surface of activated macrophages
Dr. J. Archambeau	TMEM214: a new player in the regulation of ER morphology

Poster Session#2	
Dr. D. Ricci	The sensor IRE1 couples stress detection to protein synthesis
K. Bakambamba	Control of the secretion machinery by IRE1 in human glioblastoma
Dr. X. Guillory	Targeting the IRE1 pathway for adjuvant therapies in cancers
Dr. J. Groenendyk	Loss of the Fructose Transporter SLC2A5 inhibits cancer cell migration
Dr. H. Issaoui	UPR activation in cancer cells promotes anti‐cancer immune response
S. Le Goupil	Characterization of the IRE1 BioID proximitome that controls metastatic melanoma migration and invasion
M. Nivet	A novel IRE1 target ITGA6 controls glioblastoma aggressiveness
Dr. D. Pelizzari‐Raymundo	A novel blood brain barrier‐permeable IRE1 kinase inhibitor for adjuvant glioblastoma treatment in mice
